# Necrology

**Published:** 1895-09

**Authors:** 


### NECROLOGY.

Dr. John Hazzard Records, of Little Rock, Ark., was found dead in his office in July last, having been but slightly ill the evening before. He was twenty-eight years of age, a graduate of the Veterinary Department of the University of Pennsylvania, Class of 1891, originally from Lewes, Del. He practised in Nashville, Tenn., Dallas, Texas, and recently at Little Rock.

Dr. Joseph F. Autenreith, of Jersey City, N. J., a graduate of the American Veterinary College, Class of 1882, died in July, after a lingering illness. He was a steadfast, earnest student at college, and these qualities he carried into his daily life as a practitioner. Officially he was connected with the Board of Health and Society for the Prevention of Cruelty to Animals of Jersey City, and a member in good standing of the U.S V M.A.

On September 9, 1895, at Cincinnati, O., from an overdose of morphia, Dr. Lawrence A. Anderson passed away at the age of forty-six years. A graduate of the Ontario Veterinary College, Class of 1893. He had established a stock-farm and veterinary hospital at Woodlawn, O., and later on a hospital in Cincinnati. He took a great interest in race-horses and dogs, and was the owner of the famous trotting-dog, "Jeff." Threatened blindness was the cause of his resorting to relief through morphia.

